# The Role of Erythropoietin in Bovine Sperm Physiology

**DOI:** 10.3390/ani14152175

**Published:** 2024-07-26

**Authors:** Vasiliki G. Sapanidou, Byron Asimakopoulos, Theodoros Lialiaris, Sophia N. Lavrentiadou, Konstantinos Feidantsis, Georgios Kourousekos, Maria P. Tsantarliotou

**Affiliations:** 1 Laboratory of Animal Physiology, School of Veterinary Medicine, Faculty of Health Sciences, Aristotle University of Thessaloniki, 54124 Thessaloniki, Greece; slavrent@vet.auth.gr; 2 Laboratory of Physiology, Faculty of Medicine, School of Health Science, Campus-Dragana, Democritus University of Thrace, 68100 Alexandroupolis, Greece; basima@med.duth.gr; 3 Laboratory of Genetics, Faculty of Medicine, School of Health Science, Campus-Dragana, Democritus University of Thrace, 68100 Alexandroupolis, Greece; lialiari@med.duth.gr; 4 Department of Fisheries & Aquaculture, School of Agricultural Sciences, University of Patras, 26504 Mesolonghi, Greece; kfeidant@upatras.gr; 5 Directorate of Veterinary Centre of Thessaloniki, Department of Reproduction and Artificial Insemination, National Ministry of Rural Development and Food, 57008 Thessaloniki, Greece; kourousekos@gmail.com

**Keywords:** bovine spermatozoa, erythropoietin, apoptosis, capacitation, Bcl-2, Bax, caspases

## Abstract

**Simple Summary:**

Research efforts over the past decade have uncovered new insights into functions of erythropoietin that point to new roles beyond erythropoiesis. Recent studies have demonstrated that erythropoietin is implicated in tissue protection, regeneration, and reproduction. In this context, the present study reveals for the first time that erythropoietin inhibits cell death by apoptosis and enhances the fertilizing capacity of bovine spermatozoa.

**Abstract:**

Erythropoietin (EPO), a hormone secreted mainly by the kidney, exerts its biological function by binding to its cell-surface receptor (EpoR). The presence of EPO and EpoR in the male and female reproductive system has been verified. Therefore, some of the key properties of EPO, such as its antioxidant and antiapoptotic effects, could improve the fertilizing capacity of spermatozoa. In the present study, the effect of two different concentrations of EPO (10 mIU/μL and 100 mIU/μL) on bovine sperm-quality parameters was evaluated during a post-thawing 4-h incubation at 37 °C. EPO had a positive effect on sperm motility, viability, and total antioxidant capacity. Moreover, EPO inhibited apoptosis, as it reduced both BCL2-associated X apoptosis regulator (Bax)/B-cell lymphoma 2 (Bcl-2) ratio and cleaved cysteine-aspartic proteases (caspases) substrate levels in a dose-dependent manner. In addition, EPO induced sperm capacitation and acrosome reaction in spermatozoa incubated in capacitation conditioned medeia. These results establish a foundation for the physiological role of EPO in reproductive processes and hopefully will provide an incentive for further research in order to fully decipher the role of EPO in sperm physiology and reproduction.

## 1. Introduction

Erythropoietin (EPO) is the main hormone that stimulates erythropoiesis [1]. It is a glycosylated peptide produced in the kidneys of adult mammals. The biological role of EPO is exerted through a high-affinity transmembrane receptor (EpoR) [2]. Studies have shown that EPO and its receptor are present in various non-hematopoietic tissues and organs, including the brain, the heart, and the reproductive organs of both sexes [3]. This widespread distribution of both EPO and its receptor suggests a paracrine function of EPO [4], mediating diverse effects, including its antioxidant and antiapoptotic properties [5,6,7,8]. This antiapoptotic role of EPO and the molecular mechanism(s) implicated have been widely studied [5,6]. However, the role of EPO in the reproductive system and its effect on sperm physiology in particular, are less known.

The EPO/EpoR system seems to play an interesting biological role in the reproductive system [9]. The expression of EPO mRNA has been detected in rat Sertoli cells [4], in mouse epididymis [10]), and in the human cervix, endometrium, and ovaries [11]. EPO has been identified in human seminal plasma [12], while EpoR is found in spermatozoa [13]. Interestingly, EPO plays a multifaceted role. In rats, EPO increases testosterone secretion through a specific receptor-mediated mechanism [14]. Moreover, in the same species, a long-acting EPO analogue protects spermatozoa from oxidative stress induced by doxorubicin [15].

We can speculate that the presence of EPO and/or EpoR in the testes, seminal plasma, and spermatozoa [4,12,13] creates a system with a direct effect of EPO on sperm physiology. This suggests an impact of EPO on sperm quality, fertility, and reproductive health. Indeed, in humans, EPO enhanced sperm quality by increasing sperm motility and viability [13,16]. To date, the effect of EPO on the signaling pathways of capacitation and intrinsic apoptotic cascade is still unknown. Although these two pathways represent opposite ends of a metabolic spectrum driven by oxidative promoters [17], the participation of other factors that are present in the genital tract cannot be excluded.

While much of the evidence on the role of EPO in sperm physiology relates to humans and laboratory animals, only a few studies have examined the effect of EPO on the spermatozoa of domestic animals, mainly boars and rabbits [18,19]. No data currently exist on the role of EPO on the bovine reproductive system and bull spermatozoa. Despite the differences between the bovine and human reproductive systems, the former has been used extensively to develop and optimize various technologies applied in Assisted Reproductive Technologies (ART) in humans. Both species share similarities in hormonal regulation and reproductive processes, so studies in cattle, which are free from the ethical concerns associated with human research, can provide insights into human ART procedures.

The aim of the present study was to investigate the effect of EPO supplementation on bovine spermatozoa. In particular, the effect of EPO on sperm-quality characteristics, the anti-apoptotic role, and the induction of acrosome reaction under capacitating conditions was investigated. This research aims in providing data that elucidate the role of EPO on reproductive physiology and allow us to further understand the molecular mechanisms modulated by this hormone.

## 2. Materials and Methods

### 2.1. Semen Samples

Frozen semen, collected with the use of an artificial vagina, from five mature bulls of proven fertility was kindly offered by the Center of Artificial Insemination of Thessaloniki, Greece (LicenseNo EL54SB01). The samples have been collected between November 2022 and January 2023. The personnel were trained and qualified for the procedures of semen collection. Only high-quality semen (>85% motility, <4% abnormalities, and >70% viability) was used. Semen was cryopreserved with a Tris-egg yolk extender (20% Tris-egg yolk, 7% glycerol, 78 mM citric acid, 69 mM fructose, 50 μg/mL tylosin, 250 μg/mL gentamycin, 150 μg/mL lincomycin, and 300 μg/mL spectinomycin) in 0.5 mL plastic straws (paillettes) at a concentration of 50 × 10^6^ spermatozoa/mL. Frozen samples were thawed by immersion in water (37 °C, 40 s) and pooled together. The gradual increase in temperature allowed spermatozoa to thaw slowly and regain their motility and viability.

Spermatozoa were washed three times with Sperm Tyrode’s Albumin Lactate Pyruvate (TALP) solution (100 mM NaCl, 3.1 mM KCl, 25 mM NaHCO_3_, 0.29 mM NaH_2_PO_4_, 21.6 mM sodium Lactate, 2 mM CaCl_2_, 1.5 mM MgCl_2_, 10 mM Hepes sodium salt, supplemented with 1 mM sodium pyruvate, and 50 μg/mL gentamycin) and centrifuged at 300× *g* for 10 min (25 °C). After each centrifugation, the supernatant was carefully removed, and the sperm pellet was resuspended in 1 mL Sperm TALP to repeat the process again. The concentration of spermatozoa in the final suspension was determined using a haemocytometer (OptikLabor, Grale HDS, New South Wales, Australia). Spermatozoa (50 × 10^6^ cells/mL sperm TALP) were divided into three tubes. One tube served as a negative control, while the others were supplemented with 10 mIU/μL and 100 mIU/μL epoetin-alpha (Binocrit, Sandoz Gmbh, Langkampfen, Austria), respectively.

Six experiments were performed (*n* = 6), and all assays described below were conducted in triplicates.

### 2.2. Motility Assessment

Motility of spermatozoa was determined by Computed Assisted Sperm Analyzer (CASA) using the Integrated Semen Analysis System Software (ISAS MvCo, Valencia, Spain). Five μL aliquot of sperm suspension were placed on a pre-warmed slide, and motility was determined at two different time points (0 min, 240 min) by assessing nine different parameters (Rapid, Medium, Slow, Static, Progressive Motility, Curvilinear Velocity (VCL), Straight Line Velocity (VSL), Average Path Velocity, (VAP), Amplitude Lateral Head (ALH)). The default settings were as follows: image capture by 60 frames/second, total of 25 frames; minimum contrast of 80 and medium cell size of five pixels. Progressive cell cutoff: 50 μm/sec for VAP and 80% for progressive straightness. Static cells VAP <5 μm/s, slow VAP 5–10 μm/s, and medium with a VAP 10–50 μm/s. 

### 2.3. Viability Assessment

Spermatozoa prepared as described above were smeared on glass slides and were stained with eosin/Y-nigrosin. Two hundred spermatozoa per slide were examined on an optical microscope (×100) to evaluate viability. Eosin stains dead spermatozoa pink to pale purple as the plasma membrane is permeable, while nigrosin acts as a counterstain to highlight live spermatozoa that exclude the stain.

### 2.4. Determination of Total Antioxidant Capacity (TAC)

The total antioxidant capacity of spermatozoa was determined by a 2,2-Diphenyl-1-picrylhydrazyl radical (DPPH•) scavenging assay [20]. This method is based on the elimination of the stable free radical DPPH•. Antioxidants react with DPPH•, which is reduced to DPPH-H. By accepting hydrogen, the solution loses the characteristic deep-purple color and the discoloration (lower absorbance) is proportional to scavenging capacity of the compound. Spermatozoa (5 × 10^6^) in TAC Phosphate Buffer (10 mM KH_2_PO_4_, 10 mM Na_2_HPO_4_, pH 7.4) were subjected to two cycles of sonication at 28 kHz for 60 s. Subsequently, DPPH• (0.08 mM) was added, and the samples were incubated at room temperature(RT), in the dark, for 60 min. The absorbance was measured at 517 nm, and TAC was expressed as percent inhibition of the DPPH• in relation to the absorbance of the control (100%).

### 2.5. Quantification of the Superoxide Anion (O_2_^−^) Production (Nitroblue Tetrazolium Test-NBT)

A pool of freshly thawed spermatozoa was layered onto a Percoll gradient (45% and 80%) and centrifuged (380× *g*, 25 min, RT) to remove the cryoprotectants. The supernatant was carefully removed, and the pellet was washed twice with 2 mL of Sperm TALP (140× *g*, 10 min, RT). Subsequently, spermatozoa (5 × 10^6^) were treated for 240 min in the presence or absence (negative control) of 10 or 100 mIU/μL of EPO. By the end of each incubation, EPO was removed by centrifugation (140× *g*, 10 min, RT), 6 μΜ of nitroblue-tetrazolium 2,20-bis(4-Nitrophenyl)-5,50-diphenyl-3,30-(3,30-dimethoxy-4,40 diphenylene ditetrazolium chloride) in PBS were added to each sample and incubated for 60 min at 37 °C in the dark. At the end of the incubation, the residual NBT solution was discarded by centrifugation, leaving a pellet containing the cells and blue formazan crystals, which were formed by the reduction of NBT by superoxide anion. The cells and the formazan crystals were dissolved in 120 μL of 2 M KOH and 140 μL of Dimethyl sulfoxide (DMSO). A 96-well microplate (BioTek EL800, Thomas Scientific, Swedesboro, NJ, USA) photometer was employed to measure optical density at 630 nm. Data are presented as percentage of the control sample, which was set to 100%.

### 2.6. Capacitation and Acrosome Reaction

Spermatozoa prepared as described in Section 2.5 were resuspended in IVF TALP medium (114 mM NaCl, 3.2 mM KCl, 0.34 mM NaH_2_PO_4_, 0.5 mM CaCl_2_, 10 mM Na lactate, 10 mg/mL phenol red, 30 μΜ penicillamine, 15 μΜ hypotaurine, 1 μM epinephrine supplemented with 10.4 mM pyruvate, and 50 μg/mL gentamycin in water for embryo transfer) and were incubated for 240 min at 37 °C in a 5% CO_2_ incubator in the presence (positive control) or absence (negative control) of the capacitating factor heparin (10 μg/mL) or in the presence of 10 or 100 mIU/μL EPO. Subsequently, spermatozoa were exposed to calcium ionophore (10 μM in DMSO) at 37 °C for 60 min. Calcium ionophoreinduces acrosome reaction only in capacitated spermatozoa. The samples were subsequently fixed in 4% paraformaldehyde (110 mM Na_2_HPO_4_, 2.5 mM NaH_2_PO_4_, 4% paraformaldehyde, pH 7.4) for 10 min at 25 °C. Fixing solution was removed and cells were washed twice with 100 mM ammonium acetate (pH 9.0). Twenty-five μL of each sperm suspension were smeared on glass microscope slides and air-dried. Capacitated, acrosome-reacted cells were stained with a Coomasie Blue solution (0.22% Coomassie Blue G-250, 50% methanol, 10% glacial acetic acid in distilled water) for 2 min. Slides were dried and stained, and capacitated spermatozoa were evaluated under microscopic examination (100×). The results were expressed as % of live, acrosome-reacted spermatozoa.

### 2.7. Western Blot Analysis

Percol gradient-isolated spermatozoa (Section 2.3), incubated for 240 min in the absence (negative control) or presence of EPO (10 mIU/μL and 100 mIU/μL), were homogenized (1/3 *w*/*v*) in cold lysis buffer [20 mM β-glycerophosphate, 50 mM NaF, 2 mM ethylenediaminetetraacetic acid (EDTA), 20 mM Hepes, 0.2 mM Na_3_VO_4_, 10 mM benzamidine, pH 7, 200 μM leupeptin, 10 μΜ trans-epoxy succinyl-L-leucylamido-(4-guanidino)butane, 5 mM dithiotheitol (DTT), 300 μΜ phenyl methyl sulfonyl fluoride (PMSF), 50 μg/mL pepstatin, and 1% *v*/*v* Triton X-100]. After homogenization, samples were extracted on ice for 30 min, and then they were centrifuged (10,000× *g*, 10 min, 4 °C). The BioRad protein assay was employed in order to determine protein concentrations in the samples.The resulting supernatants were boiled (3/1 *v*/*v*) with sample buffer, which contained the following ingredients: 330 mM Tris-HCl, 13% *v*/*v* glycerol, 133 mM DTT, 10% *w*/*v* Sodium Dodecyl Sulfate (SDS), and 0.2% *w*/*v* bromophenol blue). 

For the determination of Bax, Bcl-2, and β-actin levels in the samples, well-established SDS-PAGE/immunoblot analysis protocols were employed (see Figures S1 and S2). For this reason, equivalent protein amounts of 50 μg were separated on slab gels consisting of 10% *w*/*v* acrylamide and 0.275% *w*/*v* bisacrylamide. Thereafter, the separated proteins were electrophoretically transferred onto 0.45 μm nitrocellulose membranes (Schleicher and Schuell, Keene, NH, USA). For the determination of cleaved caspase levels in the samples, these were diluted to a concentration of 5 μg/mL in a 150 mM NaCl saline solution. Thereafter, 100 μL sample volumes were loaded onto a pre-soaked 0.45 μm nitrocellulose membrane (Schleicher and Schuell, Keene N. H. 03431, USA) in a dot blot vacuum apparatus (BioRad, Hercules, CA, USA), and gravity-fed through the membrane.

Then, 5% *w*/*v* non-fat milk in Tris-buffered saline with Tween (TBST), which contained 20 mM Tris-HCl, pH 7.5, 137 mM NaCl, 0.1% (*v*/*v*) Tween 20, was employed for 30 min at room temperature in order for non-specific binding sites on the membranes to be blocked. Thereafter, the nitrocellulose membranes were subjected to overnight incubation with the following antibodies, which were diluted as recommended by the manufacturer’s guidelines: anti-Bcl2 (Cell Signaling, Beverly, MA, USA), anti-Bax (B-9) (Cell Signaling, Beverly, MA, USA), and Cleaved Caspase Substrate (Cell Signaling, Beverly, MA, USA). Quality transfer and protein loading were assured by β-actin (Cell Signaling, Beverly, MA, USA).

After washing in TBST (3 periods, 5 min each time), the blots and dots were incubated with horseradish peroxidase-linked secondary antibodies and washed again in TBST (3 periods, 5 min each time). Thereafter, enhanced chemiluminescence (Chemicon, Tokyo, Japan) with exposure to Fuji Medical X-ray films was employed to detect bands. Finally, laser-scanning densitometry was used to quantify the dark bands that correspond to the protein bands on the films (GelPro Analyzer Software, GraphPad, version 10.0).

### 2.8. Statistical Analysis 

The data are presented as mean ± SD. The results were analyzed using SPSS (version 22.0). Repeated measures Analysis of Variance (ANOVA) with the Bonferroni correction was used for the statistical analysis, where the interaction between different concentrations of EPO and the two time points was analyzed. A value of *p* < 0.05 was considered statistically significant.

## 3. Results

### 3.1. EPO Preserved the Motility of Spermatozoa 

The results from the motility assessment are presented in Table 1 and Table 2. EPO had no effect on most of the CASA kinematic parameters. However, a statistically significant higher percentage of rapid spermatozoa (37.17%) was observed (Table 1) after 240 min of incubation in the presence of 100 mIU/μL EPO (*p* = 0.037). In particular, the motility of control spermatozoa was reduced by almost 50% during the 240 min incubation period. However, EPO at 10 or 100 mIU/μL significantly decreased this reduction to 37.64% and 10.86%, respectively. The interaction between treatment and time is statistically significant (*p* = 0.002). Specifically, the percentage of the reduction was 49.65%, 37.64%, and 10.86% for the control, EPO10, and EPO100 groups, respectively.

### 3.2. EPO Had a Positive Effect on Sperm Viability 

**Figure 1:**
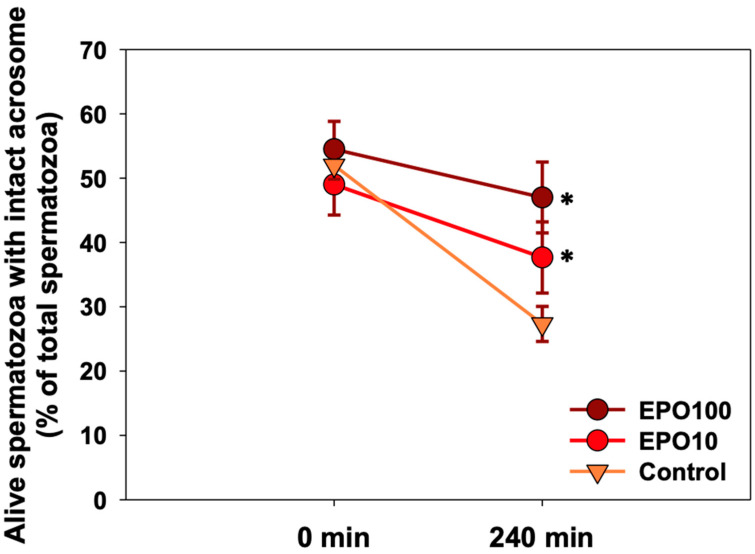
The effect of EPO (10 mΙU/μL and 100 mIU/μL) supplementation on the percentage of live spermatozoa with intact acrosome during a 240 min incubation. Data are presented as mean ± SD. Asterisk (*) depicts statistically significant difference between the control and the treated groups for each given time point (*p* < 0.05, *n* = 6).

Figure 1 shows the results regarding the evaluation of viability. The viability of spermatozoa in the control group was reduced, and this effect was directly related to the duration of the incubation period *(p* = 0.002). At 240 min of incubation, both concentrations of EPO showed a higher percentage of live spermatozoa with intact acrosome compared to the control group (Figure 1, *p* < 0.05). No statistically significant differences were observed among EPO concentrations.

### 3.3. EPO Showed a Trend to Reduce O_2_^−^

**Figure 2:**
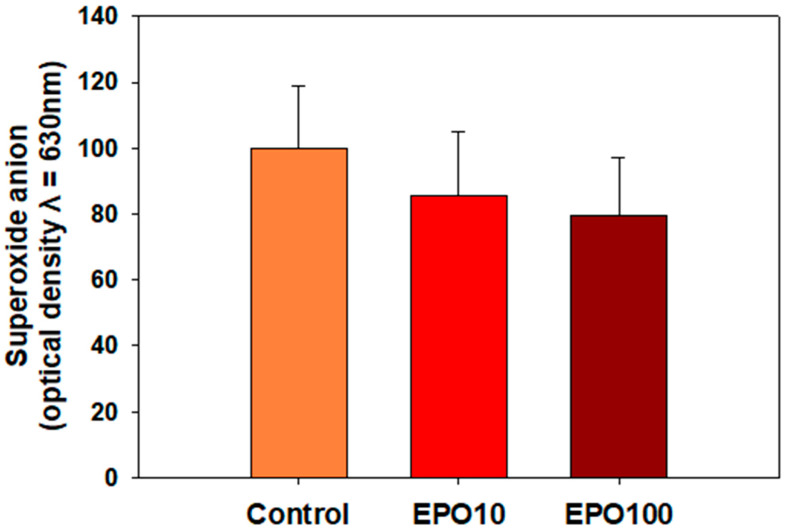
Intracellular superoxide production of frozen/thawed bovine spermatozoa supplemented with two different concentrations of EPO (10 mΙU/μL and 100 mIU/μL) after 240 min incubation in the presence of a negative control, which was set as 100%. Data are presented as mean ± SD (*p* < 0.05, *n* = 6).

No change in intracellular O_2_^−^ levels was observed under these experimental conditions (Figure 2). However, in the presence of EPO, there was a moderate, although not statistically significant, decrease in O_2_^−^ levels (*p* = 0.058).

### 3.4. ΕΡO. Augmented the Total Antioxidant Capacity (TAC) of Spermatozoa

**Figure 3:**
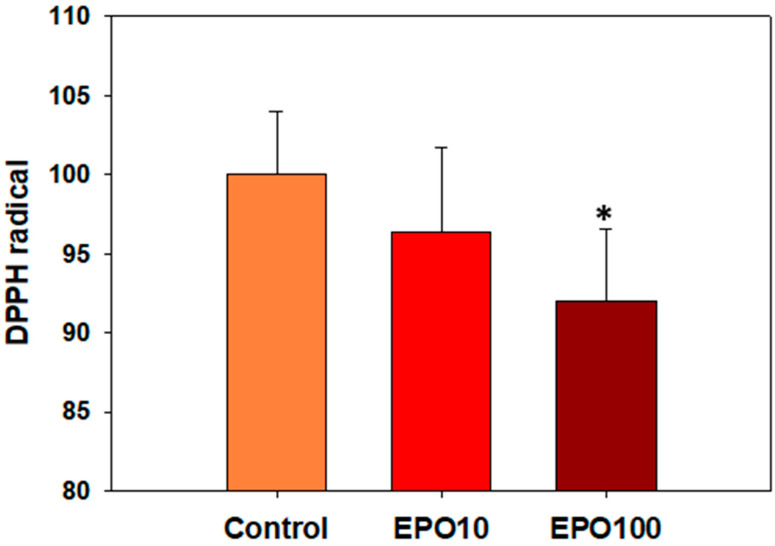
Total antioxidant capacity determined as % of reduced DPPH^•^ in frozen/thawed bovine spermatozoa supplemented with two different concentrations of EPO (10 mΙU/μL and 100 mIU/μL) after 240 min incubation in the presence of a negative control, which was set as 100%. Data are presented as mean ± SD. Asterisk (*) denotes statistically significant differences (*p* < 0.05) compared to the control group (*p* < 0.05, *n* = 6).

The percentages of DPPH• reduction were 3.69% and 8.02% for 10 mIU/μL and 100 mIU/μL EPO, respectively. The higher concentration of EPO significantly reduced the DPPH• compared to the control group (Figure 3, *p* < 0.05).

### 3.5. EPOInduced Capacitation and Acrosome Reaction in Spermatozoa Under Capacitating Conditions

**Figure 4:**
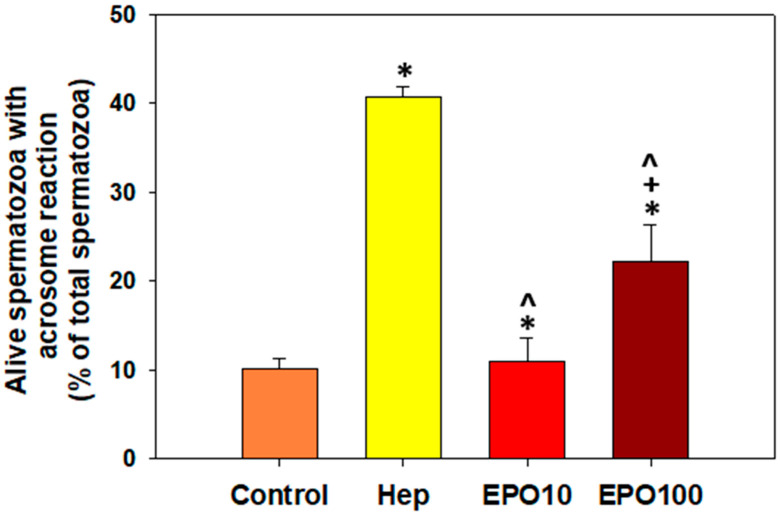
The effect of EPO (10 mΙU/μL and 100 mIU/μL) supplementation on the percentage of live spermatozoa with acrosome reaction. Data are presented as mean ± SD. Asterisk (*) denotes statistically significant differences (*p* < 0.05) compared to the control group, cross (+) denotes statistically significant differences between the two concentrations of EPO, while caret (^) indicates statistically significant differences compared to the positive control (heparin) (*p* < 0.05, *n* = 6).

Figure 4 illustrates the results of the evaluation of acrosome-reacted spermatozoa upon EPO supplementation/the effect of EPO on the induction of acrosome reactions under capacitating conditions. The supplementation of EPO (100 mIU/μL) in the appropriate buffer triggered the capacitation of spermatozoa (22.2% acrosome reacted cells) in the presence of calcium ionophore. EPO mimicks a typical capacitating factor, heparin, which was used as a positive control (Figure 4, yellow bar). Specifically, EPO (100 mIU/μL) increased the percentage of capacitated, acrosome-reacted spermatozoa to 22.2%, compared to the negative control (10.2%) and the lower EPO concentration (10.91%) (*p* < 0.05). Heparin induced capacitation to 40.7% of the spermatozoa, a 4-fold increase compared to the negative control.

### 3.6. EPOInhibited Apoptosis in Spermatozoa

**Figure 5:**
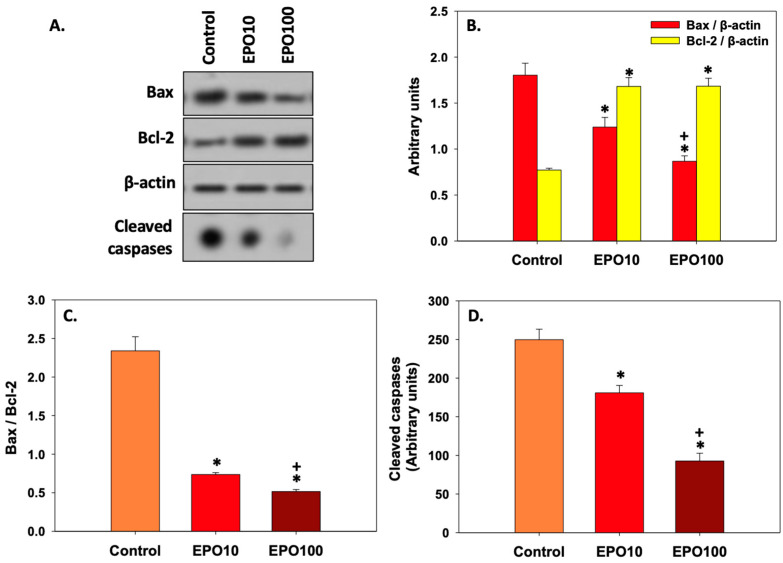
Analysis of apoptotic markers Bax, Bcl-2 (normalized to β-actin), and cleaved caspases, as shown by representative blots (**A**). Bax, Bcl-2 (**B**), Bax/Bcl-2 ratio (**C**), and cleaved caspase (**D**) levels of frozen/thawed bovine spermatozoa under the effect of 10 mIU/μL (ΕPO10) and 100 mIU/μL (ΕPO100) EPO. Spermatozoa extracts from control, Epo 10, and Epo 100 groups were immunoblotted for Bax, Bcl-2, cleaved caspases, and β-actin to verify equal loading. Asterisk (*) denotes statistically significant differences (*p* < 0.05) compared to the control group, while cross (+) denotes statistically significant differences (*p* < 0.05) between the two concentrations of EPO (Panels B, C, and D). Values constitute means ± SD (*n* = 6).

Figure 5 shows the effect of EPO on the expression of specific apoptosis-related markers. In Panel A, the protein levels of both Bax and Bcl-2 under the effect of EPO treatment are shown. While Bcl-2 levels significantly increased (*p* < 0.05) in the presence of either concentration of EPO compared to untreated control cells, no statistically significant differences were observed in Bcl-2 levels between spermatozoa treated with different concentrations of EPO. On the other hand, Bax levels decreased significantly in spermatozoa exposed to EPO compared to the control (*p* < 0.05). In addition, Bax levels were significantly lower (*p* < 0.05) in spermatozoa treated with 100 mIU/μL EPO than in spermatozoa treated with 10 mIU/μL EPO (Panel B).

When Bax/Bcl-2 ratio (Panel C) was considered, statistical differences were observed not only between the EPO-treated and the control groups, but also among the two EPO-treated groups. Interestingly, the Bax/Bcl-2 ratio and activated caspases (Panel D) were decreased almost 3-fold in the treated groups compared to the control group, thus indicating a more pronounced anti-apoptotic effect of EPO on bovine spermatozoa.

## 4. Discussion

The EPO/EpoR system plays an interesting biological role in haemopoietic and non-hemopoietic tissues [3]. The role of this system in the genital tract of both sexes seems to open a new field of research in reproductive physiology. In the current study, we investigated for the first time the antioxidant and antiapoptotic effect of EPO on bovine spermatozoa during a 240-min incubation. Two concentrations of EPO have been tested based on preliminary data from our research group but also from other studies [13,16]. EPO induced capacitation/acrosome reaction and improved the viability of frozen/thawed bovine spermatozoa, probably due to the inhibition of apoptosis. EPO increased only the percentage of rapid spermatozoa.

Our results showed that EPO had a moderate effect on superoxide anion levels, which was statistically significant. Different mechanisms have been proposed to support the antioxidant role of EPO, such as the regulation of gene expression of antioxidant enzymes and the regulation of mitochondrial function [5,21]. It has been suggested that EPO upregulates the expression of antioxidant enzymes, such as Superoxide Dismutase (SOD) and Catalase (CAT), to prevent oxidative damage in rats [7]. Finally, EPO has been shown to reduce mitochondrial ROS production [21] to ensure redox homeostasis and prevent oxidative damage. In the present study, the antioxidant role of EPO has been approached from two perspectives. EPO supplementation did not affect the levels of superoxide anion. Although the NBT test that was applied here can provide valuable information about the sperm oxidative status of the cells, we should not rule out that this is due to the sensitivity limitations of the assay and/or the experimental conditions. On the other hand, the antioxidant capacity of spermatozoa, as determined by the TAC assay, was increased by EPO (100 mIU/μL) after 240 min of incubation. This assay reflects the ability of all antioxidants present in a biological system to counteract oxidation. In other words, TAC is a sum of the antioxidant activities of a biological system [20]. In conclusion, EPO augments the antioxidant capacity of spermatozoa, but more sophisticated techniques could verify the effect of EPO on sperm oxidative status since sperm physiology is affected by different types of ROS, H_2_O_2,_ and NO.

In contrast to what has been observed in human sperm [13,16], no beneficial effect on bovine sperm motility was identified. Asimakopoulos and co-authors suggested that EPO at the concentration of 100 mIU/μL increased the percentage of spermatozoa with progressive motility compared to the control group, while at a lower concentration (10 mIU/μL), it mediated no effect [13]. Interestingly, in our study incubation of spermatozoa with 100 mIU/μL, EPO for 240 min increased the percentage of spermatozoa with rapid movement compared to the corresponding control cells. In our study, the effect of EPO has been tested in frozen/thawed spermatozoa, which, due to the freeze/thawing process, faces the challenge of osmotic and cold shock. This can partially explain why EPO has a more pronounced effect on fresh-ejaculated human sperm [13,16]. However, the positive effect of EPO at this concentration on rapid motility implies that EPO, at this concentration at least, may induce the phosphorylation of axonemal proteins and, therefore, maintain the percentage of rapid spermatozoa, probably through the engagement of a series of signaling pathways [22].

Τhe percentage of live spermatozoa was preserved during the 240-min incubation due to the supplementation of the media with EPO. A similar effect was also observed in human spermatozoa [16]. The positive effect of EPO on the viability of spermatozoa could be exerted through the modulation of apoptotic mechanisms [22]. The role of EPO in the apoptotic cascade of spermatozoa has not been described before. However, it seems that this effect is modulated by the biological consequences of EPO binding to its receptor. The above hypothesis has been verified by the results of the present study since EPO dose-dependently inhibited apoptotic events, as indicated by the decrease of the Bax/Bcl-2 ratio and cleaved caspases, compared to the control group. The Bax/Bcl-2 ratio can act as a rheostat, which determines cell susceptibility to apoptosis [23]. In line with this observation, cleaved caspases, the main units of the apoptotic machinery, are significantly decreased dose-dependently due to EPO supplementation. Consequently, the present study shows that EPO inhibits key targets of the apoptotic cascade (caspases and pro-apoptotic Bax) and upregulates the expression of the anti-apoptotic Bcl-2 [5,6]. However, further research could investigate the role of specific caspases, such as caspase-3,8,9, as well as the existence of EpoR in bovine spermatozoa.

In contrast with previous studies, which did not detect Bax in bovine spermatozoa [24,25], more recent studies like the one of Baňas and co-authors [26], as well as the present study, confirm the expression of this protein in bovine spermatozoa. This observation can be attributed to the improvement of the specificity and sensitivity of the antibody–antigen interaction.

Finally, as mentioned before, EPO at the concentration of 100 mIU/μL of EPO acted as a capacitating factor, resulting in an increased percentage of bovine spermatozoa with acrosome reaction. This process is very complex and involves changes in sperm membrane properties, motility patterns, and biochemical modifications [27]. Bovine spermatozoa typically require at least 240 min to capacitate a process that enables spermatozoa to fertilize the oocyte [28]. The presence of the EpoR in bovine spermatozoa and the activation of protein kinases, which phosphorylate tyrosine residues and phospholipase C, seems more than likely [29]. It is highly possible that EPO acts as a heparin-binding protein that is mainly produced by the accessory glands of the male under the control of androgens and, in this context, favors capacitation and maintains cellular homeostasis [30]. Conclusively, we could speculate that the effect of EPO on capacitation/acrosome reaction does not seem to be related to the modulation of ROS concentrations, even though this role should be further investigated. However, it is also possible that EPO modulates other metabolic pathways, such as those that result in the phosphorylation of axonemal proteins and increased intracellular concentration of Ca^2+^ and cAMP [31]. We anticipate that this study will provide an incentive for future studies to elucidate these mechanisms.

## 5. Conclusions

The present study reveals a protective (antioxidant/antiapoptotic) role of EPO on bovine frozen/thawed spermatozoa through an enhancement of sperm total antioxidant capacity and an ameliorative effect on sperm rapid motility and viability. A new role for EPO is suggested, which is its triggering effect on sperm capacitation/acrosome reaction. The latest is supported by findings that EPO exists in the female reproductive tract and may influence sperm fertilizing capacity. Further research is needed to investigate the efficacy of EPO in protecting the quality of cryopreserved sperm and to determine which molecular pathways/ligands and genes EPO activates for a better understanding of its role in the fertilization process and male infertility.

## Figures and Tables

**Table 1:** The effect of EPO (10 mΙU/μL and 100 mΙU/μL) on motility parameters of frozen/thawed bovine spermatozoa during a 240 min incubation in the presence of a negative control. Data are presented as mean ± SD. Asterisk (*) indicates statistically significant difference compared to the control groups for each particular time point (*p* < 0.05, *n* = 6).

Min	Group	Rapid (%)	Medium (%)	Slow (%)	Total Motile (%)	Static (%)	Progressive Motile (%)
0	Control	43.1±2.75	21.46 ± 2.65	5.26 ± 2.65	69.82 ± 7.80	30.18 ± 7.80	21.05 ± 3.29
	EPO 10	45.48 ± 2.77	25.68 ± 4.66	8.1 ± 6.04	79.26 ± 13.40	20.74 ± 12.23	20.51 ± 3.69
	EPO 100	41.7 ± 3.16	22.44 ± 6.96	15.57 ± 15.87	79.67 ± 7.88	20.33 ± 3.75	20.8 ± 3.75
240	Control	21.7 ± 3.16	22.44 ± 6.96	15.57 ± 15.87	59.71 ± 6.30	40.29 ± 6.30	20.8 ± 3.75
	EPO 10	28.36 ± 4.45	25.00 ± 10.02	8.65 ± 3.15	62.01 ± 11.51	37.99 ± 11.53	24.05 ± 3.07
	EPO 100	37.17 ± 2.3 *	19.67 ± 6.77	6.55 ± 2.79	63.39 ± 6.71	36.61 ± 6.71	24.70 ± 3.45

**Table 2:** The effect of EPO (10 mΙU/μL and 100 mIU/μL) on CASA kinematic parameters of frozen/thawed bovine spermatozoa, after a 240 min incubation, in the presence of a negative control. Data are presented as mean ± SD (*p* < 0.05, *n* = 6).

Min	Group	VCL (μm/s)	VSL(μm/s)	VAP (μm/s)	ALH
0	Control	59.43 ± 5.78	19.7 ± 2.95	33.36 ± 2.73	3.00 ± 0.34
	EPO 10	58.76 ± 4.76	19.08 ± 1.30	32.66 ± 1.73	3.05 ± 0.33
	EPO 100	59.06 ± 5.07	18.85 ± 2.65	32.96 ± 4.24	3.26 ± 0.53
240	Control	53.75 ± 10.51	24.78 ± 7.73	34.92 ± 7.10	2.42 ± 0.22
	EPO 10	52.51 ± 7.13	23.46 ± 4.61	27.81 ± 10.29	2.95 ± 0.38
	EPO 100	56.95 ± 8.77	30.55 ± 6.74	40.28 ± 7.11	2.28 ± 0.36

## Data Availability

The data presented in this study are available on request from the corresponding authors.

## Supplementary Materials

The following supporting information can be downloaded at: https://www.mdpi.com/article/10.3390/ani14152175/s1. Figure S1: The complete original immunoblots shown in Figure 5 regarding Bax, Bcl-2 and β-actin are presented in order below. The individual parts comprising Figure 5 are specified using black boxes; Figure S2: The complete original immunoblots shown in Figure 5 regarding cleaved caspases are presented in order below. The individual parts comprising Figure 5 are specified using black boxes.
