# Supplementary material for: The Role of Erythropoietin in Bovine Sperm Physiology
Source: Animals (Basel). 2024 Jul 26;14(15):2175. doi: 10.3390/ani14152175 (PMC11311055; doi:10.3390/ani14152175)

The role of erythropoietin in bovine sperm physiology

Vasiliki G. Sapanidou[1], Byron Asimakopoulos[2], Theodoros Lialiaris[3], Sophia N. Lavrentiadou[1], Konstantinos Feidantsis[4], Georgios Kourousekos[5], Maria P. Tsantarliotou[1, *]

[1] Laboratory of Physiology, School of Veterinary Medicine, Faculty of Health Sciences, Aristotle University of Thessaloniki, University Campus, 54124 Thessaloniki, Greece.

[2] Laboratory of Physiology, Faculty of Medicine, School of Health Science, Democritus University of Thrace, University Campus-Dragana, 68100 Alexandroupolis, Greece.

[3] Laboratory of Genetics, Faculty of Medicine, School of Health Science, Democritus University of Thrace, University Campus-Dragana, 68100 Alexandroupolis, Greece.

[4] Department of Fisheries & Aquaculture, School of Agricultural Sciences, University of Patras, 26504 Mesolonghi, Greece

[5] Directorate of Veterinary Centre of Thessaloniki, Department of Reproduction and Artificial Insemination, National Ministry of Rural Development and Food, 57008, Ionia, Thessaloniki, Greece.

Figure S1: The complete original immunoblots shown in Figure 5 regarding Bax, Bcl-2 and β-actin are presented in order below. The individual parts comprising Figure 5 are specified using black boxes.

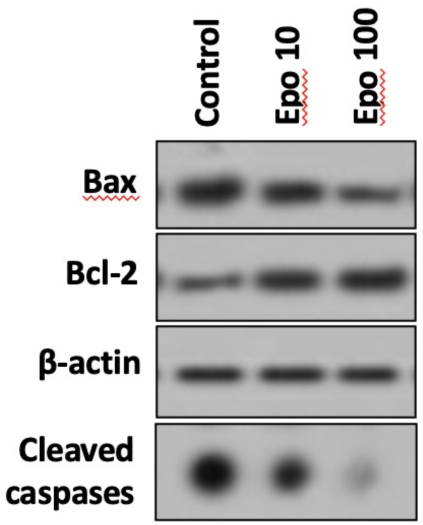

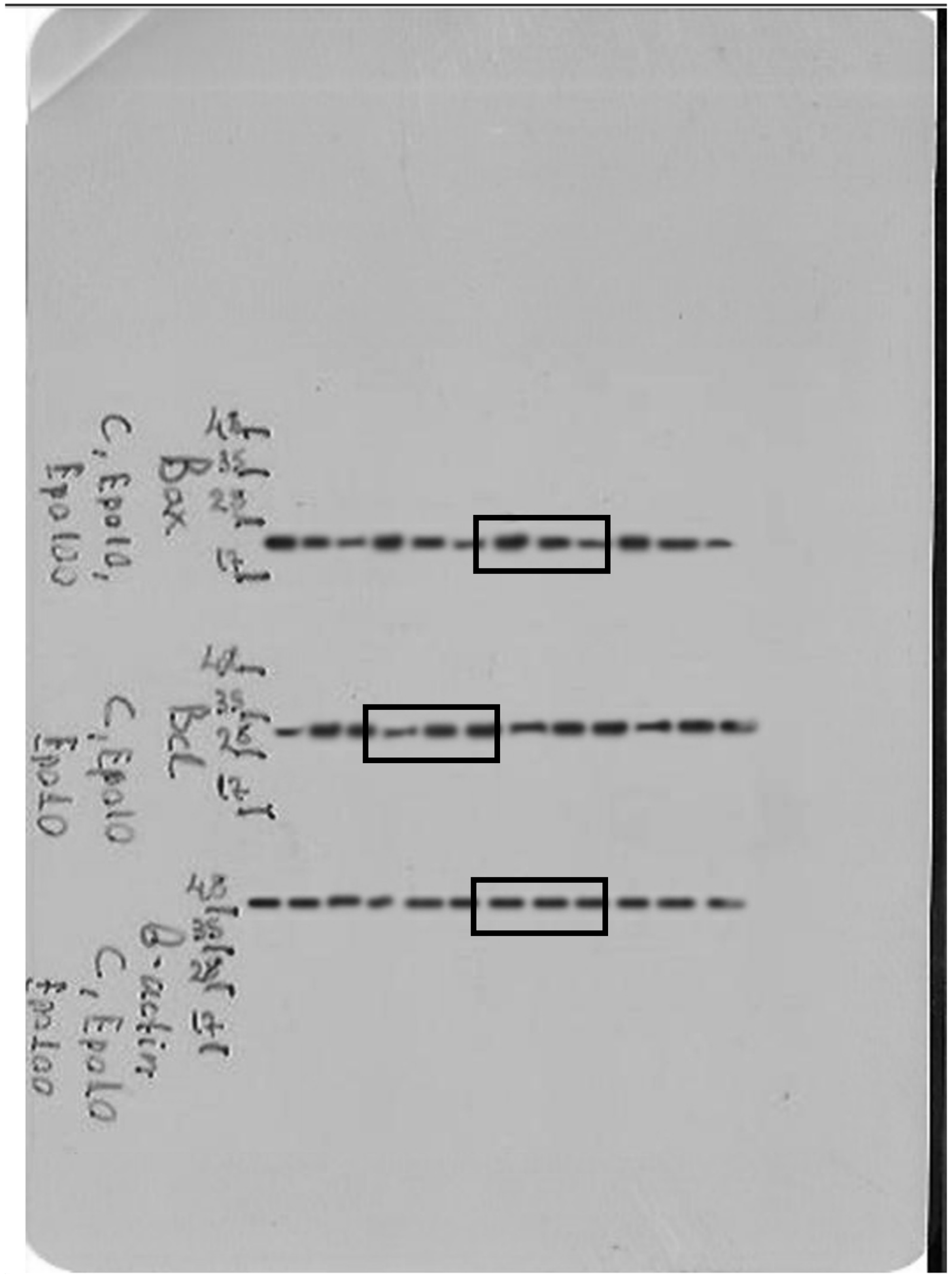

Figure S2: The complete original immunoblots shown in Figure 5 regarding cleaved caspases are presented in order below. The individual parts comprising Figure 5 are specified using black boxes.

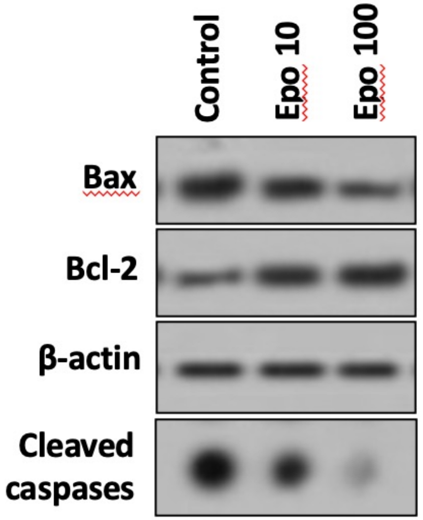

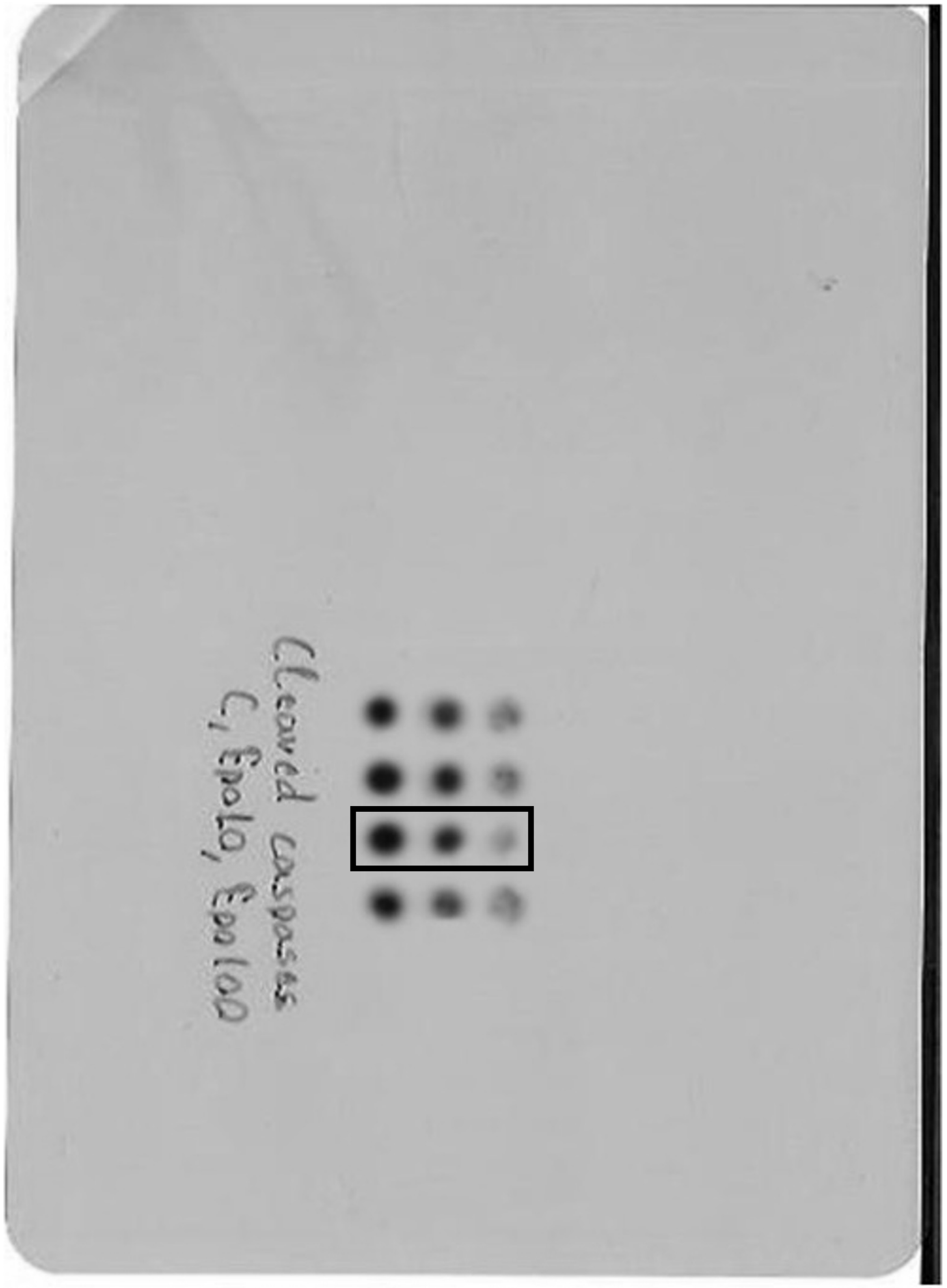

Supplement: Supplementary file 1 [file animals-14-02175-s001.zip › animals-3103222-supplementary.pdf]
